# Prevalence and Influencing Factors of Overweight and Obesity Among Left-Behind Children Under 6 Years Old in China: A Cross-Sectional Study

**DOI:** 10.3390/nu18010079

**Published:** 2025-12-26

**Authors:** Zhaoyang Fan, Jing Nan, Chen Zhou, Dongmei Yu, Shuya Cai, Ruilian Wang, Yuxiang Yang, Liyun Zhao, Yuying Wang

**Affiliations:** 1 Capital Center for Children’s Health, Capital Medical University, Capital Institute of Pediatrics, Beijing 100020, China; fanzhaoyang@mail.ccmu.edu.cn; 2 National Institute for Nutrition and Health, Chinese Center for Disease Control and Prevention, Beijing 100050, China; nj13939012762@163.com (J.N.); zc983702782@163.com (C.Z.); caisy@ninh.chinacdc.cn (S.C.); yangyuxiang1996@sina.com (Y.Y.); zhaoly@ninh.chinacdc.cn (L.Z.)

**Keywords:** overweight, obesity, left-behind children

## Abstract

**Objectives**: To analyze the prevalence and influencing factors of overweight and obesity among left-behind children (LBC) under 6 years old in China, and to provide a reference basis for their early prevention and control. **Methods**: The data were derived from the National Nutrition and Health Survey among children and lactating mothers (2016–2017). A total of 19,229 left-behind children under 6 years old in China were included in this study. The results were post-stratification weighted and adjusted using data from the Sixth National Population Census released by the National Bureau of Statistics of China in 2010. The Rao–Scott chi-square test with sampling design-weighted correction was used to test for statistical differences, and multivariate unconditional logistic regression analysis was conducted to explore influencing factors. **Results**: The prevalence of overweight and obesity among LBC under 6 years old in China were 6.68% and 2.22%, respectively. The overweight rate and obesity rate of boys were higher than those of girls (7.96% vs. 5.15%, 2.77% vs. 1.56%). Both the overweight rate and obesity rate showed a “U”-shaped trend with increasing age (*p* < 0.0001). LBC with migrant fathers had the highest overweight rate and obesity rate. Logistic regression analysis indicated that being male, being in infancy or preschool age, residing in eastern China, having a migrant father, and higher annual per capita household income were risk factors for overweight and obesity. **Conclusions**: Left-behind children under 6 years old in China are at risk of overweight and obesity. Among LBC under 6 years old in China, the issues of overweight and obesity are relatively prominent in boys, as well as those in infancy and preschool age. Additionally, LBC with fathers who migrate for work have relatively higher overweight/obesity rates. It is essential to pay attention to the problems of overweight and obesity among LBC under 6 years old in China, strengthen the monitoring of their growth and development, and incorporate the improvement of overweight and obesity in LBC into national nutrition improvement policies at all levels.

## 1. Introduction

With the advancement of China’s new-type urbanization drive, the issue of Left-behind children (LBC) has become a prominent social problem [1]. According to the data from the seventh national population census, it is estimated that among children in China in 2020, the proportion of left-behind children aged 0–17 was 22.5% [2]. Annual Report on Chinese Children’s Development (2024) shows that nearly half of the children nationwide were affected by population mobility in 2020 [3], while the latest data from the Ministry of Civil Affairs indicates that the number of LBC in China reached over 9.02 million in 2023, still maintaining a large scale [4]. The healthy growth of LBC is influenced by various factors such as genetic factors, environment, and economic level, and they face many uncertainties [5]. On one hand, parents’ migration for better employment opportunities may be accompanied by higher income, which in turn can provide LBC with more abundant material reserves and a wider range of health services, improving their growth environment and quality of life to a certain extent [6]; on the other hand, due to the lack of parental care and supervision, the weakening of family care capacity, and the unbalanced distribution of corresponding resources such as food and medical care, LBC are more prone to nutritional and health problems [7]. Currently, the health status of LBC in China presents a “health paradox” where undernutrition and overnutrition (including overweight and obesity) coexist [8].

Studies have shown that overweight and obesity during the preschool years have a significant impact on children’s physical and mental health as well as their future development [9], and this is also true for children living in rural areas. In the long run, being overweight or obese may increase the potential risk of chronic diseases such as obesity, diabetes, and cardiovascular diseases for preschool children when they grow up [10]. Overweight and obesity among LBC are a prominent issue in childhood malnutrition in China [11,12], which not only affects children’s current growth and development but also impacts the health quality of the future population and the quality of the labor force through intergenerational transmission, posing an undeniable public health challenge [13].

With economic development and shifting cultural norms, spatial mobility within China’s population is poised to remain on an upward trajectory [2]. Consequently, the phenomenon of left-behind children is likely to become increasingly prevalent, and this group will emerge as a vital component of China’s future labor force. Enhancing their physical health holds profound significance for facilitating China’s transition from a populous nation to one with robust human capital. Therefore, investigating the nutritional and health issues of preschool left-behind children, adjusting relevant policies in a timely manner, and safeguarding their well-being are of paramount importance for the nation’s long-term development.

However, current social research on overweight and obesity among preschool LBC is limited [13], and insufficient attention has been paid to the existence of LBC in urban areas. To address this gap, this study uses representative data from the National Nutrition and Health Survey among children and lactating mothers to analyze the epidemiological characteristics and influencing factors of overweight and obesity among LBC under 6 years old nationwide. It also clarifies the current prevalence of this condition in the target group and proposes targeted recommendations. In doing so, this research aims to provide robust empirical evidence to effectively address the challenges associated with LBC and support the formulation and refinement of relevant policies.

## 2. Materials and Methods

### 2.1. Data Source

The data were derived from the National Nutrition and Health Survey among children and lactating mothers (2016–2017), a component of the China National Nutrition and Health Surveillance (2015–2017). For this round of surveillance, a multistage stratified cluster sampling method was adopted. This method considered the balanced distribution of stratified factors such as region and urban-rural areas, while also taking into account the existing work foundation and conditions. Ultimately, 275 monitoring sites were identified across 31 provinces/autonomous regions/municipalities in mainland China to conduct nutrition surveys on children and lactating mothers. A previous study has detailed information concerning the study design, sampling method, and quality control process [14].

**Figure 1:**
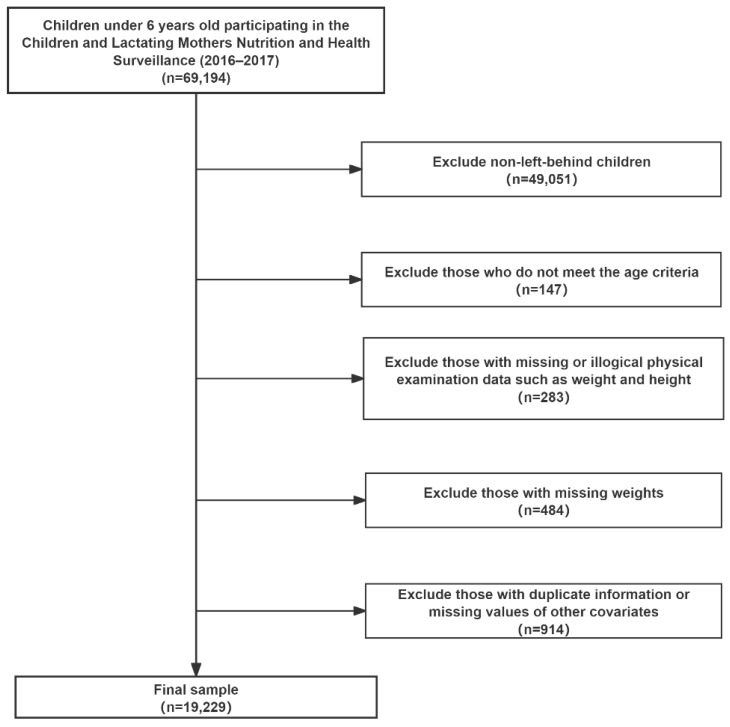
Flowchart of research sample screening process.

In this study, LBC were defined as children whose one or both parents migrated for work in various regions. Children under 6 years old with complete basic information who participated in physical examinations were included; those with abnormal height and weight data, duplicate data, or missing values of other important variables (such as age, gender, areas, region, parents’ education level and the types of parental migration for work) were excluded. Finally, a total of 19,229 valid samples of LBC under 6 years old in China (2016–2017) were obtained (Figure 1).

This project was reviewed and approved by the Ethics Committee of the Chinese Center for Disease Control and Prevention (Protocol Code: 201614, 3 June 2016). Before the survey, the guardian of each participant signed an informed consent form on their behalf. The data have national and provincial representativeness.

### 2.2. Data Collection

The National Nutrition and Health Survey among children and lactating mothers (2016–2017) collected information through four parts: questionnaire survey, physical examination, dietary evaluation, and laboratory test. The questionnaire survey and physical examination were mainly used in this study.

The questionnaire survey employed a standardized instrument designed by the national project team. Uniformly trained investigators collected basic information from parents or guardians through face-to-face interviews. The questionnaire covered personal details, family characteristics, and household economic status.

Physical examination was performed by investigators using a unified method for centralized measurement. For children aged 0~2 years, recumbent length was measured with an infantometer to the nearest 0.1 cm. For children aged 2~5 years, standing height was measured using a metal stadiometer, also accurate to 0.1 cm. Weight for all children aged 0–5 years was measured with an electronic scale calibrated to 0.1 kg.

### 2.3. Definition of Overweight and Obesity

Overweight and obesity in children under 6 years of age were defined according to the World Health Organization (WHO) child growth standards [15]. Based on the child’s age in months, sex, weight, and recumbent length (for children <2 years) or standing height (for children ≥2 years), Z-scores were calculated to assess nutritional status.

For children aged 0–4 years, the WHO 2006 Growth Standards were applied. Overweight was defined as a weight-for-height Z-score (WHZ) >2 and ≤3, and obesity as a WHZ > 3. For 5-year-old children, the WHO 2007 Growth References were used, with overweight defined as a body mass index-for-age Z-score (BMIZ) >1 and ≤2, and obesity as a BMIZ > 2.

### 2.4. Covariates

A wide range of potential confounders were accounted for: (1) Gender included male and female. (2) The age group of this study is divided into six groups (years): 0~, 1~, 2~, 3~, 4~, 5~6. (3) Areas: including megacities, small and medium-sized cities, general rural areas and impoverished rural areas [16]. (4) Region: divided into eastern, central and western [17]. (5) Parents’ education level: divided into low (primary school and below), medium level (junior high school) and high level (high school/secondary school/technical school and above). (6) Annual household per capita income: divided into low, medium and high levels according to the interquartile range. The low-level income was <6000 yuan, the middle-level income was 6000–15,000 yuan, and the higher-level income was ≥15,000 yuan. The unreported were divided into another group. (7) Household annual dietary expenditure: according to the quartile spacing, it is divided into low, medium and high levels. The low-level expenditure was less than 10,000 yuan, the medium-level expenditure was 10,000–20,000 yuan, and the higher-level expenditure was more than 20,000 yuan. The unreported were divided into another group. (8) The types of parental migration for work are categorized into three groups: both parents migrating for work, mother migrating for work, and father migrating for work.

### 2.5. Quality Control

From 2016 to 2017, the nutrition and health monitoring of children and lactating mothers in China strictly followed the principles of unified program, manual and questionnaire, unified training and assessment, unified equipment and reagents, and unified data entry and cleaning for quality control. The quality control for height and weight measurement was carried out at both provincial and national levels. The results indicated that the coincidence rates of height and weight obtained by provincial quality control personnel were 98.5% and 91.5%, respectively. The corresponding agreement rates of height and weight obtained by national quality control personnel were 95.7% and 88.2%, respectively. These high consistency rates support the reliability and validity of the anthropometric data collected.

### 2.6. Statistical Analysis

SAS 9.4 and R4.4.0 software were used for data cleaning and analysis. SURVEYFREQ was used to calculate the rate of overweight and obesity in children, and Rao–Scott chi-square test based on weighted correction of sampling design was used for statistical difference test. According to the data of children in the sixth census of the National Bureau of Statistics in 2010, the post-hierarchical weight adjustment was carried out. The final complex sampling weight was calculated by multiplying the basic sampling weight and the post-hierarchical adjustment weight. Multivariate unconditional logistic regression model was used to analyze the influencing factors of overweight and obesity in LBC under 6 years old. A two-sided *p*-value < 0.05 was defined as statistical significance.

## 3. Results

### 3.1. General Characteristics of the Participants

A total of 19,229 LBC under 6 years old in China were included in the analysis, with 9593 (49.89%) boys and 9636 (50.11%) girls. Among them, 608 (3.16%) were from megacities, 3421 (17.79%) from medium and small cities, 10,340 (53.77%) from general rural areas, and 4860 (25.27%) from impoverished rural areas. In terms of geographic regions, the samples of LBC from eastern, central, and western China were 5470 (28.45%), 7602 (39.53%), and 6157 (32.02%), respectively (Table 1).

### 3.2. Overweight and Obesity Among LBC with Different Characteristics

The standardized overweight rate of LBC under 6 years old in China was 6.68%. Stratified by gender, the rate was 7.96% for boys and 5.15% for girls. By residential area, overweight rates were 4.99% in megacities, 7.26% in small and medium-sized cities, 6.57% in general rural areas), and 6.64% in impoverished rural areas. Among age groups, the rates were 10.77% (0-year-olds), 8.28% (1-year-olds), 4.62% (2-year-olds), 3.90% (3-year-olds), 2.51% (4-year-olds), and 11.75% (5-year-olds), respectively. Geographically, overweight rates were 7.10% in eastern China, 7.04% in central China, and 5.69% in western China.

Statistically significant differences in LBC’s overweight rate were observed across genders (*p* < 0.0001), age groups (*p* < 0.0001), and annual household dietary expenditure levels (*p* = 0.0198). Specifically, boys showed a higher overweight rate than girls; the highest rates were found among 0-year group, 5-year group, and the group with low annual household dietary expenditure (Table 2).

The standardized obesity rate of LBC under 6 years old in China was 2.22%. Stratified by gender, the rates were 2.77% for boys and 1.56% for girls. By region, obesity rates were 2.41% in eastern China, 2.16% in central China, and 2.09% in western China. According to residential area type, the rates were 1.73% in megacities, 1.80% in small and medium-sized cities, 2.20% in ordinary rural areas, and 2.50% in impoverished rural areas. Among age groups (0–5 years), obesity rates were 4.15% for 0-year-olds, 1.44% for 1-year-olds, 1.04% for 2-year-olds 1.38% for 3-year-olds, 0.98% for 4-year-olds, and 4.95% for 5-year-olds, respectively. Statistically significant differences in obesity rates were observed across genders (*p* < 0.0001) and age groups (*p* < 0.0001): boys had a higher obesity rate than girls, and the highest rates were found in 0-year-old and 5-year-old groups (Table 3).

### 3.3. Influencing Factors of Overweight and Obesity in LBC

Multivariate unconditional logistic regression analysis was performed with gender, age, place of residence, region, type of parental migration, annual household per capita income, annual household dietary expenditure, and parental education level as independent variables, and whether LBC under 6 years old in China were overweight or obese as dependent variables. A stepwise regression method was used for variable selection, and the final results are presented in Table 4.

After adjusting for other factors, the results indicated that girls had a significantly lower risk of overweight and obesity compared to boys (OR = 0.69, 95% CI = 0.62–0.76). Compared with the 0-year-old group, the risk of overweight and obesity in the 1–4-year-old group gradually decreased [OR (95% CI): 0.58 (0.50–0.68), 0.34 (0.28–0.41), 0.30 (0.25–0.37), 0.23 (0.18–0.28)], while 5-year-olds showed a significantly higher risk (OR = 1.16, 95% CI = 1.01–1.32). LBC living in the central and western regions [OR (95% CI): 0.86 (0.77–0.97), 0.73 (0.64–0.83)] had a significantly lower risk of overweight and obesity than those in the eastern region. Compared with migrant parents, migrant fathers were a risk factor for overweight and obesity in LBC (OR = 1.21, 95% CI = 1.07–1.36). Furthermore, compared to the low-income group, middle (OR = 1.28, 95% CI: 1.02–1.47) and high (OR = 1.36, 95% CI: 1.08–1.71) income levels were associated with an increased risk. No significant associations were found between overweight/obesity and place of residence, annual household per capita income, or parental educational level among LBC.

## 4. Discussion

The problem of LBC is a stage problem in the process of China’s economic and social development. The growth and health status of LBC has become a hot issue of concern [18]. Previous studies have confirmed that left-behind children have malnutrition problems such as growth retardation, low weight and anemia [19,20]. However, with the changes in dietary structure and lifestyle in China, the problem of overweight and obesity is becoming more and more obvious in LBC [11,21]. Overweight and obesity can greatly increase the risk of serious health damage in children, and also damage the individual’s mental and mental health [22,23].

This study, utilizing nationally representative data, found that the standardized overweight and obesity rates among Chinese LBC under 6 years old were 6.68% and 2.22%, respectively. Key determinants identified included gender, age, geographical region, parental migration type, and household economic status. Given the significant health risks associated with childhood obesity, these findings highlight specific, modifiable factors that should be prioritized in targeted prevention strategies for this vulnerable population.

Results from the 2013 China Nutrition and Health Surveillance of Children Aged 0–5 Years and Lactating Mothers showed that the prevalence of overweight and obesity among LBC under 6 years old in rural China were 6.9% and 2.3% [11], respectively. These figures are comparable to the findings of the present study, indicating that the obesogenic environment for Chinese LBC persists. Thus, preventing overweight and obesity in LBC remains a critical issue for ensuring children’s healthy growth. However, comparisons with several regional studies reporting higher rates must be interpreted cautiously [24]. These discrepancies may stem from differences in the operational definition of LBC, the age ranges studied, and, importantly, the sampling framework. Unlike surveys focused solely on rural settings, our data encompass a broader socioeconomic spectrum, which may yield a more conservative national estimate.

Notably, the risk of overweight and obesity exhibited a U-shaped curve across age groups, peaking in infancy (0-year-olds) and the pre-school period (5-year-olds). This pattern aligns with the general trajectory of childhood adiposity development in China [25], which may be attributed to the fact that infancy and the pre-school period are peak stages for adipose tissue development. The peak at age 5 may reflect increased dietary autonomy and potential exposure to energy-dense foods within the left-behind environment [25]. Furthermore, being male was a consistent risk factor, with boys having significantly higher prevalence and odds compared to girls. This is corroborated by prior research [19,26] and may be attributed to both higher physiological energy requirements and sociocultural feeding practices that favor greater food intake for boys [27,28]. The study results showed that the urban-rural difference in overweight and obesity among LBC was no longer significant, indicating that the problem of overweight and obesity among rural LBC is increasingly severe. This finding is consistent with the research by Wu [13]. However, differences in the prevalence of overweight and obesity among LBC persisted across regions with different levels of economic development, suggesting that the macro-level socioeconomic development exerts a non-negligible impact on childhood malnutrition [26,27]. Additionally, studies have revealed that regional differences in children’s health status are prominent, and regional factors contribute the most to disparities in children’s health in China [28]—a conclusion that aligns with the result of the present study, where the prevalence of overweight and obesity in the eastern region were higher than those in the central and western regions.

There is an inseparable link between human health and economic income [3]. Compared with the low-income group, medium and high income was a risk factor for overweight in LBC. In general, the consumption level of a household is largely determined by its income, and household income can fundamentally reflect the standard and quality of life [29]. Studies on the income and dietary composition of Chinese residents have shown that high-income residents in China have a higher proportion of high-fat and high-protein foods in their diets. On one hand, higher income can improve the dietary quality of LBC; on the other hand, it may lead to excessive nutrition due to over-care from caregivers and unreasonable dietary intake, thereby increasing the prevalence of overweight and obesity [18]. Overall, household economic income may have a dual impact on children’s dietary behaviors, suggesting that caregivers of LBC from high-income households need to rationalize dietary expenditures and foster the development of healthy dietary habits in children. Compared with the scenario where both parents migrate for work, father-only migration was a risk factor for overweight and obesity in LBC. This could be linked to remittances improving food affordability without concurrent improvements in dietary knowledge or caregiving practices [9], a hypothesis warranting qualitative exploration.

This study has certain limitations: (1) Based on a cross-sectional design, this study cannot reflect the dynamic changes in the prevalence of overweight and obesity among the study participants, nor can it establish a causal relationship between overweight/obesity and its influencing factors. In future research, cohort studies could be integrated to further confirm the changing trends in the prevalence of overweight and obesity among Chinese preschool LBC and identify their influencing factors. (2) This study did not consider the impact of dietary intake on overweight and obesity in LBC. Future research could explore the dietary status of this population to analyze the causal relationships between nutritional status and factors such as dietary patterns and dietary behaviors in LBC.

Nevertheless, this study also has certain innovations: It utilizes recent, high-quality national surveillance data, providing a representative picture of preschool LBC in China. By including urban LBC, it expands the scope beyond the typical focus on rural settings. The findings pinpoint specific demographic and socioeconomic subgroups at elevated risk, offering concrete targets for public health intervention.

## 5. Conclusions

Based on nationally representative data, this study examined the prevalence and determinants of overweight and obesity among left-behind children under six years of age in China. The analysis identified several key modifiable risk factors, including male sex, specific age groups (0- and 5-year-olds), residence in the eastern region, paternal migration, and higher household income. These findings underscore the importance of early and targeted interventions aimed at these factors for the primary prevention of overweight and obesity in this vulnerable population. The evidence provided here contributes to a focused public health strategy to improve nutritional outcomes and long-term health in early childhood.

## Figures and Tables

**Table 1:** Sample composition of LBC * under 6 years in China, 2016–2017.

Characteristics	Male		Female		Total	
	*N*	%	*N*	%	*N*	%
Age group (years)						
0~	2157	22.49	2219	23.03	4376	22.76
1~	1375	14.33	1362	14.13	2737	14.23
2~	1488	15.51	1411	14.64	2899	15.08
3~	1499	15.63	1499	15.56	2998	15.59
4~	1560	16.26	1592	16.52	3152	16.39
5~	1514	15.78	1553	16.12	3067	15.95
Region						
Megacities	308	3.21	300	3.11	608	3.16
Small and medium-sized cities	1687	17.59	1734	18.00	3421	17.79
General rural	5161	53.80	5179	53.75	10,340	53.77
Impoverished rural	2437	25.40	2423	25.15	4860	25.27
Geographical area						
Eastern	2725	28.41	2745	28.49	5470	28.45
Central	3789	39.50	3813	39.57	7602	39.53
Western	3079	32.10	3078	31.94	6157	32.02
Parents’ migration types						
both parents migrated for work	3200	33.36	3157	32.76	6357	33.06
father migrated for work	6029	62.85	6135	63.67	12,164	63.26
mother migrated for work	364	3.79	344	3.57	708	3.68
Annual per capita income						
Low	1270	13.24	1282	13.30	2552	13.27
Medium	2504	26.10	2578	26.75	5082	26.43
High	1190	12.40	1198	12.43	2388	12.42
Not provided	4629	48.25	4578	47.51	9207	47.88
Annual meal expenditure						
Low	1805	18.82	1758	18.24	3563	18.53
Medium	1698	17.70	1792	18.60	3490	18.15
High	1236	12.88	1234	12.81	2470	12.85
Not provided	4854	50.60	4852	50.35	9706	50.48
Mother’s education level						
Low	1482	15.45	1439	14.93	2921	15.19
Medium	7025	73.23	7083	73.51	14,108	73.37
High	1086	11.32	1114	11.56	2200	11.44
Father’s education level						
Low	1232	12.84	1209	12.55	2441	12.69
Medium	7218	75.24	7275	75.50	14,493	75.37
High	1143	11.91	1152	11.96	2295	11.94

* LBC: Left-behind children.

**Table 2:** Prevalence of overweight among LBC * under 6 years in China, 2016–2017 (%, 95% CI).

Characteristics	*N*	Overweight			*Rao–Scott χ* ^2^	*p*
		%	95% *CI*			
Total	1284	6.68	5.96	7.41	-	-
Gender					30.6579	<0.0001
Male	723	7.96	6.91	9.01		
Female	561	5.15	4.48	5.82		
Age group (years)					120.349	<0.0001
0~	461	10.77	9.16	12.39		
1~	196	8.28	6.55	10.01		
2~	119	4.62	3.12	6.13		
3~	97	3.90	2.29	5.51		
4~	85	2.51	1.84	3.18		
5~	326	11.75	9.94	13.56		
Region					0.7494	0.8615
Megacities	52	4.99	1.77	8.22		
Small and medium-sized cities	227	7.26	5.37	9.15		
General rural	729	6.57	5.69	7.44		
Impoverished rural	276	6.64	5.07	8.21		
Geographical area					3.3702	0.1854
Eastern	410	7.10	5.96	8.24		
Central	516	7.04	5.64	8.44		
Western	358	5.69	4.81	6.58		
Parents’ migration types					10.3798	0.0056
both parents migrated for work	336	5.78	4.83	6.72		
father migrated for work	912	7.32	6.49	8.15		
mother migrated for work	36	5.00	2.69	7.32		
Annual per capita income					5.7141	0.1264
Low	142	5.62	4.18	7.05		
Medium	338	6.45	5.44	7.46		
High	168	5.86	4.42	7.30		
Not provided	636	7.22	6.24	8.20		
Annual meal expenditure					9.8567	0.0198
Low	225	6.83	5.44	8.22		
Medium	242	6.09	5.00	7.18		
High	147	4.91	3.67	6.15		
Not provided	670	7.24	6.27	8.21		
Mother’s education level					3.2322	0.1987
Low	182	5.89	4.87	6.91		
Medium	946	6.65	5.88	7.43		
High	156	8.12	5.39	10.84		
Father’s education level					3.5112	0.1728
Low	168	6.45	5.16	7.73		
Medium	958	6.44	5.66	7.23		
High	158	8.65	5.47	11.82		

* LBC: Left-behind children.

**Table 3:** Prevalence of obesity among LBC * under 6 years in China, 2016–2017 (%, 95% CI).

Characteristics	*N*	Obesity			*Rao–Scott χ* ^2^	*p*
		%	95% *CI*			
Total	459	2.22	1.82	2.62	-	-
Gender					21.1005	<0.0001
Male	284	2.77	2.25	3.30		
Female	175	1.56	1.15	1.96		
Age group (years)					119.326	<0.0001
0~	170	4.15	3.16	5.15		
1~	43	1.44	0.86	2.02		
2~	31	1.04	0.58	1.50		
3~	41	1.38	0.77	1.98		
4~	25	0.98	0.47	1.48		
5~	149	4.95	3.67	6.22		
Region					1.8245	0.6096
Megacities	13	1.73	0.20	3.25		
Small and medium-sized cities	73	1.80	0.93	2.66		
General rural	257	2.20	1.74	2.66		
Impoverished rural	116	2.50	1.60	3.40		
Geographical area					0.4411	0.8021
Eastern	163	2.41	1.64	3.18		
Central	174	2.16	1.48	2.83		
Western	122	2.09	1.47	2.72		
Parents’ migration types					3.8152	0.1484
both parents migrated for work	122	1.88	1.30	2.45		
father migrated for work	324	2.47	1.91	3.03		
mother migrated for work	13	1.39	0.35	2.44		
Annual per capita income					1.0314	0.7937
Low	49	1.87	1.20	2.54		
Medium	133	2.34	1.66	3.03		
High	69	2.39	1.61	3.16		
Not provided	208	2.20	1.64	2.76		
Annual meal expenditure					1.2317	0.7454
Low	78	1.91	1.23	2.59		
Medium	90	2.21	1.58	2.84		
High	60	2.13	1.26	3.00		
Not provided	231	2.34	1.83	2.85		
Mother’s education level					3.4749	0.176
Low	59	1.81	1.10	2.53		
Medium	346	2.20	1.73	2.66		
High	54	3.03	1.84	4.22		
Father’s education level					0.8088	0.6674
Low	58	2.55	1.57	3.54		
Medium	343	2.20	1.75	2.64		
High	58	2.01	1.17	2.85		

* LBC: Left-behind children.

**Table 4:** Multivariate unconditional logistic regression of overweight/obesity among LBC * under 6 in China, 2016–2017.

Influencing Factor		β	SE	Wald χ^2^	*p*	OR (95% CI)
Intercept		−1.62	0.08	381.27	<0.01	-
Gender	Female vs. Male	−0.37	0.05	51.61	<0.01	0.69 (0.62~0.76)
Age group (years)	1~ vs. 0~	−0.54	0.08	44.23	<0.01	0.58 (0.50~0.68)
	2~ vs. 0~	−1.08	0.10	127.18	<0.01	0.34 (0.28~0.41)
	3~ vs. 0~	−1.19	0.10	144.19	<0.01	0.30 (0.25~0.37)
	4~ vs. 0~	−1.48	0.11	188.56	<0.01	0.23 (0.18~0.28)
	5~ vs. 0~	0.15	0.07	4.41	0.04	1.16 (1.01~1.32)
Geographical area	Central vs. Eastern	−0.15	0.06	6.01	<0.01	0.86 (0.77~0.97)
	Western vs. Eastern	−0.31	0.07	22.64	<0.01	0.73 (0.64~0.83)
Parents’ migration types	Father migrated for work vs. Both parents migrated for work	0.19	0.06	9.73	<0.01	1.21 (1.07~1.36)
	Mother migrated for work vs. Both parents migrated for work	−0.03	0.16	0.03	0.86	0.97 (0.71~1.33)
Annual per capita income	Medium vs. Low	0.19	0.10	3.91	0.04	1.28 (1.02~1.47)
	High vs. Low	0.31	0.12	6.91	<0.01	1.36 (1.08~1.71)

* LBC: Left-behind children.

## Data Availability

The data presented in this study are available on request from the corresponding author. This restriction is in accordance with the regulations of the Institute of Nutrition and Health of the Chinese Center for Disease Control and Prevention, which do not permit the public disclosure of these data.

## Abbreviations

The following abbreviations are used in this manuscript:

LBC	Left-behind children
WHO	World Health Organization
WHZ	Weight for Height Z-score
BMIZ	Body Mass Index for Age Z-score
